# Liposomal Taro Lectin Nanocapsules Control Human Glioblastoma and Mammary Adenocarcinoma Cell Proliferation

**DOI:** 10.3390/molecules24030471

**Published:** 2019-01-29

**Authors:** Anna C. N. T. F. Corrêa, Mauricio A. Vericimo, Andriy Dashevskiy, Patricia R. Pereira, Vania M. F. Paschoalin

**Affiliations:** 1 Chemistry Institute, Universidade Federal do Rio de Janeiro, Rio de Janeiro 21941-909, Brazil; annac.correa@hotmail.com (A.C.N.T.F.C.); biopatbr@gmail.com (P.R.P.); 2 Immunobiology Department, Universidade Federal Fluminense, Niterói 24020-150, Brazil; vericimo@vm.uff.br; 3 Pharmaceutical Technology Department, Freie Universität Berlin, 12169 Berlin, Germany; dashevsk@zedat.fu-berlin.de

**Keywords:** *Colocasia esculenta*, food bioactive, tarin, stable nanocapsules, entrapment efficiency, no-toxicity, preclinical tests, antitumoral activity, chemotherapeutic adjuvant

## Abstract

The search for natural anticancer agents and nanocarrier uses are a part of the current strategies to overcome the side effects caused by chemotherapeutics. Liposomal nanocapsules loaded with purified tarin, a potential immunomodulatory and antitumoral lectin found in taro corms, were produced. Liposomes were composed by 1,2-dioleoyl-sn-glycerol-3-phosphoethanolamine, cholesterylhemisuccinate, and 1,2-distearoyl-sn-glycero-3-phosphoethanolamine-*N*-[folate(polyethylene glycol)-2000 prepared by thin-film hydration. Small unilamellar vesicles were achieved by sonication and extrusion. Scanning electron microscopy evidenced round-shaped nanocapsules presenting a smooth surface, 150 nm diameter and polydispersity index <0.2, estimated by dynamic light scattering. Tarin entrapment rates were over 80% and leakage of ~3% under 40 days of storage at 4 °C. Entrapped tarin exhibited an 83% release after 6 h at pH 4.6–7.4 and 36 °C. Both free and encapsulated tarin exhibited no in vitro toxicity against healthy mice bone marrow and L929 cells but stimulated the production of fibroblast-like and large round-shaped cells. Encapsulated tarin resulted in inhibition of human glioblastoma (U-87 MG) and breast adenocarcinoma (MDA-MB-231) proliferation, with an IC_50_ of 39.36 and 71.38 µg/mL, respectively. The effectiveness of encapsulated tarin was similar to conventional chemotherapy drugs, such as cisplatin and temozolide. Tarin liposomal nanocapsules exhibited superior pharmacological activity compared to free tarin as a potential chemotherapy adjuvant.

## 1. Introduction

Cancer is among the leading causes of death worldwide, defined by the exacerbated proliferation of normal cells into tumor cells due to a multistage process that usually involves mutation [1]. Due to the high number of new cancer diagnoses every year, over 4700 every day in the USA alone, many studies, including breast cancer and glioblastoma proliferation control research, are being developed in order to search for the best strategy to improve patient quality of life and life expectancy [2].

Breast cancer is the second main cause of death by cancer in women, second only to lung cancer. The American Cancer Society estimated that about 266,120 new cases of invasive breast cancer in women would be diagnosed in 2018, and that about 40,920 women would die from this disease [3]. Most women with breast cancer require surgical intervention to remove the tumor and, may, before or after, undergo radiotherapy or chemotherapy to control or avoid metastasis. Anthracyclines, such as doxorubicin (Adriamycin) and epirubicin (Ellence), taxanes, like paclitaxel (taxol) and docetaxel (taxotere), 5-fluorouracil (5-FU), cyclophosphamide (cytoxan), and carboplatin (paraplatin) are the most common drugs applied in as a two or three-combination for breast cancer treatment [4]. On the other hand, glioblastoma affects the central nervous system and represents 81% of malignant brain tumors, with a low average survival estimate of five years in 5% of all cases [5]. Temozolomide has been the most common FDA-approved glioma treatment for over 20 years [6]. However, like other chemotherapeutics, temozolomide reduces the number of circulating leukocytes, making the patient more susceptible to infections, and also leads to other side effects, such as vomiting, nausea, and alopecia. A combination therapy has been suggested to improve survival rates [7].

Throughout the years, several strategies have been used to overcome or minimize side effects, including the search for natural anticancer agents, which are extensively associated to lower toxicity, due to their favorable body absorption and metabolism compared to conventional synthetic drugs [8]. Additionally, progress in cancer therapeutics has led to the development of nanosystems, including liposomes, to increase intracellular drug concentrations in cancerous cells while minimizing toxicity to normal cells [9], a non-specific characteristic of conventional treatments. Current therapies have focused on the use of biodegradable nanoparticles to encapsulate pharmacologically active compounds that can later be released into the bloodstream or the desired local target, and have been explored as a way to protect active molecules from enzymatic degradation and increase their bioavailability.

Tarin has been purified and fully characterized as a lectin naturally found in taro corms (*Colocasia esculenta)* [10,11,12,13]. Studies on tarin biological activities have revealed that this lectin exhibits both in vitro and in vivo immunomodulatory potential, as well as promising anticancer and antimetastatic properties [10,14,15,16,17]. Although much is known about the encapsulation of bioactive compounds in liposomes and tarin bioactivities, no study has been carried out on tarin encapsulation in nanoliposomes. Tarin encapsulation into liposomes could potentiate the pharmacological properties of this molecule, as well as therapeutic treatment results, reducing the necessary dose of new compounds, decreasing side effects or undesirable interactions and allowing tarin to remain longer in the blood stream [18,19].

In this context, tarin liposomal nanocapsules were produced and characterized and the immunomodulatory and antitumoral potentials of the bioactive lectin nanocapsules were evaluated in order to apply this newly designed composite as a future chemotherapeutic adjuvant.

## 2. Results

### 2.1. Liposomal Tarin Encapsulation and Characterization

**Figure 1:**
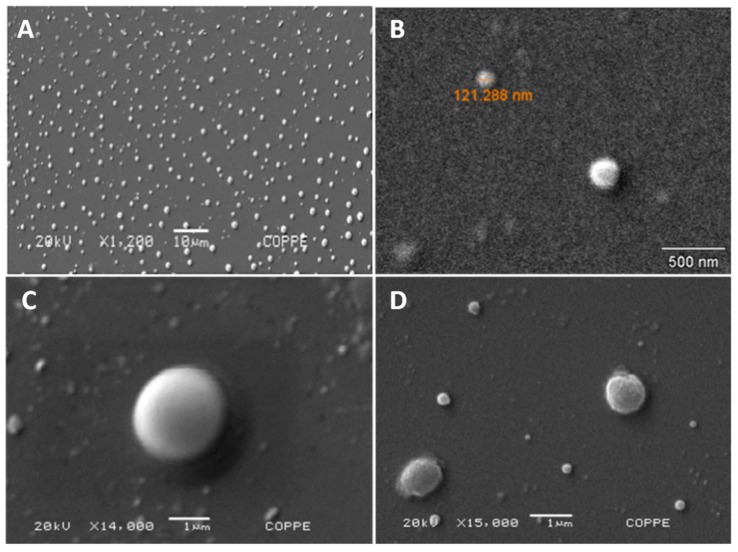
Morphological characterization of liposomal tarin nanocapsules. A scanning electron microscope was used to record DOPE, PEG, and CHEMS nanocapsules (formulation A1). Photographs show liposomes at 20 kV and magnification of 1200× (**A**); 45,000× (**B**); 14,000× (**C**) and 15,000× (**D**).

Liposomal tarin nanocapsules were prepared by an extrusion technique based on two distinct previously reported methods, reaching encapsulation efficiencies of 29% and 68%, respectively [20,21]. Scanning electron microscopy (SEM) and dynamic light scattering (DLS) analyses revealed the presence of smooth-surfaced round-shaped vesicles, with an average size of ~150 nm and polydispersity index (PdI) of 0.168 on the first day, confirming successful liposomal nanocapsule production (Figure 1 and Table 1). 

The stability of the liposomal nanocapsules (A1 formulation) under storage at 4 °C for 180 days, obtained through 0.2 μm pore membrane extrusion, was evaluated by DLS. Entrapment efficiency was determined by the ratio between the amount of unencapsulated tarin and initial tarin load [22].

**Figure 2:**
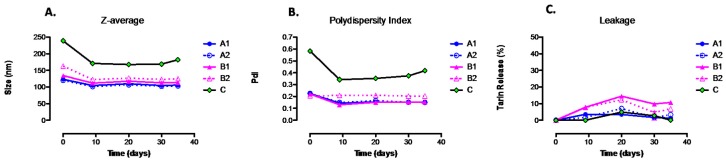
Physicochemical characterization and stability evaluation of liposomal of tarin formulations. The Z-average (**A**), polydispersity index (**B**) and leakage (**C**) of liposome preparations storage at 4 °C were monitored for 40 days. A1, A2, B1, B2, and C represent distinct liposomal encapsulation formulations, presented in Figure S1.

In order to optimize liposomal tarin encapsulation, a formulation based on the dos Santos Ferreira et al. [21] methodology was modified regarding liposome composition, sonication, tarin entrapment time, initial tarin load, and organic solvent (Figure S1). Liposomes prepared from DOPE (1,2-dioleoyl-sn-glycerol-3-phosphoethanolamine) as the fundamental component plus CHEMS and DSPE-PEG (2000), presented diameters ranging from 120 nm to 163.2 nm, and PdI close to 0.2 for the A1, A2, B1, and B2 formulations (Figure 2A,B). Nanocapsules size and polydispersity index were not significantly affected during 40 days of storage at 4 °C. On the other hand, formulation C prepared without DSPE-PEG (2000) exhibited a fluctuating PdI between 0.6 and 0.4 and diameter size (Z-average) ranging from 238.8 nm to around 170 nm when comparing day 0 to successive days (Figure 2A,B and Figure S1). 

The type of organic solvent, sonication time (1 or 10 min), initial tarin load (1 mg/mL or 2 mg/mL) and exposure time for tarin entrapment did not affect the size or homogeneity of any of the formulations (Figure 2A,B and Figure S1).

Tarin leakage assays monitored for 40 days at 4 °C storage indicate that liposome formulations with the highest tarin leakage were those prepared using methanol, namely B1 and B2 formulations, which presented 14.5 and 12.3% leakages, respectively, compared to the chloroform formulations, which exhibited less than 7% tarin leakage profiles (Figure 2C). Based on physical analyses, formulations A1 and A2 presented the most promising features and less leakage, of under 1.5% and 3.3%, respectively, combined with size stability and homogeneity during 40 days of storage at 4 °C (Figure 2A–C). Since sonication time did not lead to significant differences, formulation A1 was adopted in order to produce stable vesicles for the subsequent studies.

The stability of formulation A1 was monitored by a size distribution evaluation, Z-average and PdI during 180 days of storage at 4 °C. An extrusion membrane porosity of 0.2 μm produced liposomal nanocapsules with average size of around 150 nm and PdI from 0.135 to 0.192, which did not significantly vary during the storage period (Table 1). Tarin entrapment efficiency into liposomal nanocapsules was very high, reaching 83%. In other independent experiments, entrapment efficiency was even higher, reaching >90% and once again, PEG exhibited an important role in liposome formulation since liposomes produced without PEG exhibited less efficient tarin entrapment (79% tarin encapsulation—data not shown).

Taken together, the optimized outlined protocol for tarin encapsulation in nanoliposomes should follow the steps described for the A1 formulation and extrusion should be performed through 0.2 μm pore membranes to proceed to the in vitro and in vivo tarin liposomal nanocapsule bioactivity assays.

### 2.2. Release Control under Physiological Conditions

**Figure 3:**
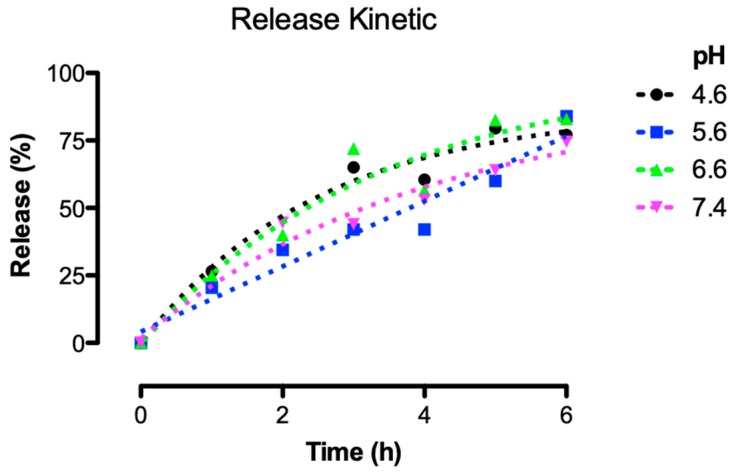
Release profile of encapsulated tarin exposed to pH 4.6, 5.6, 6.6, and 7.4, mimicking human body traits and cellular compartments. Tarin liposomal nanocapsules (A1 formulation) prepared through 0.2 μm pore extrusion membrane were maintained at 36 °C for 6 h. Tarin release was measured hourly, as described by Peterson [23].

To evaluate the performance of the liposomes formulated with DOPE, PEG, and CHEMS, dissolved in chloroform and extruded through a 0.2 μm membrane (A1 formulation), a kinetic release assay was carried out for 6 h, mimicking human body pH conditions (4.6, 5.6, 6.6, and 7.4). The liposomes produced herein were capable of releasing entrapped tarin in a controlled manner, in both acidic and neutral environments. The time necessary to release half the tarin loads at pH 4.6 and 6.6 was reached in 2.3 h, occurring between 4 and 5 h for pH 5.6 and, for pH 7.4, around 4 h. After 6 h, almost 80% of the tarin had been released at all pHs of interest (Figure 3).

### 2.3. In Vitro Pre-Clinical Tests

#### 2.3.1. Toxicological Screening and Morphological Modifications of Healthy Mice Cells Treated with Free or Encapsulated Tarin

**Figure 4:**
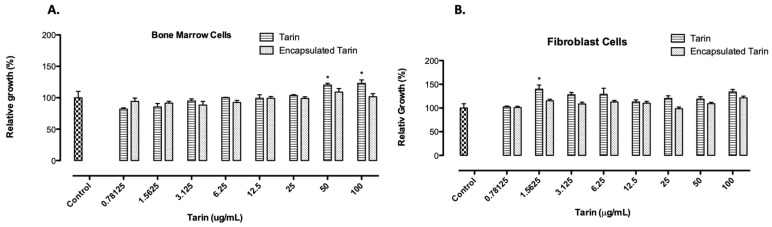
Toxicological effects of free or encapsulated tarin on healthy mice cells. (**A**) bone marrow and (**B**) fibroblast L929 cell line. Viability of cells exposed to increasing doses of free or encapsulated tarin for 24 h was determined using resazurin as indicator. Cultures with no tarin addition were used as control. * *p* < 0.05 compared to control.

The viabilities of mice bone marrow and fibroblast L929 cells cultivated in the presence of increasing free or encapsulated tarin (A1 formulation) concentrations ranging from 0.78125 to 100 μg/mL were not affected. Instead, free tarin exhibited a proliferative effect, enhancing the number of bone marrow cells with tarin at 50 or 100 μg/mL. When encapsulated tarin was tested, no dose-dependent cytotoxicity was observed for both lineage cells, reinforcing the potential applicability of liposomal tarin nanocapsules for future studies in murine models (Figure 4).

**Figure 5:**
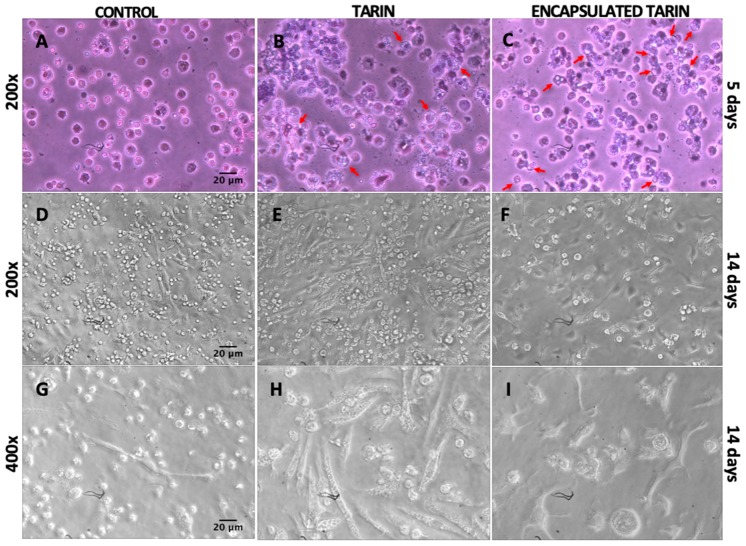
Morphological characteristics of mice bone marrow cells treated with free and encapsulated tarin. Bone marrow cells were harvested from cultures after 5 (**A**–**C**) or directly observed from cultures after 14 days (**D**–**I**). Five-day culture cells were subjected to cytospin and stained with Grunwald-Giemsa. Red arrows indicate the presence of vesicles inside the cytoplasm. Photographs were recorded at 200× and 400× magnifications.

The morphological characteristics of bone marrow cells cultured with free or encapsulated tarin (20 μg/mL) were monitored during 14 days, revealing several differences, such as alterations in cell density between control wells and between cells treated with free or encapsulated tarin (Figure 5A–I). On the fifth day, control wells displayed a considerably higher number of cells (Figure 5A–C). However, after 14 days, the cell-occupied area (61.5%) was enhanced after tarin treatment, in both free (96.8%) and encapsulated (94%) form (Figure 5D–F). The percentage of elongated cells was also increased when treated by tarin (95.5% free-tarin; 90.9% encapsulated tarin) while the percentage of occupied area remained the same and was reduced in the controls (35.6%) (control versus tarin and control versus encapsulated tarin) (Figure 5D–F).

Control cells were smaller and presented homogeneous and similar morphological characteristics, with no significant variability within the cell population (Figure 5A,D,G). However, after 14 days of treatment, tarin in its free form (Figure 5E,H) led to a high number of fibroblast-like cells, while a significant amount of large round cells was detected in wells containing encapsulated tarin, with the suggestive appearance of stromal and progenitor cells, respectively (Figure 5F,I).

Cytosmears of cultured cells revealed the presence of numerous spherical cells with prominent surface ruffles, blebs and reniform nucleus, characteristic of monocytes. Granules and numerous vesicles were also evidenced at or near the cell surface, reinforcing the hypothesis that cells exposed to tarin treatment for five days may be monocytes [24]. Multilobed nucleus cells, characteristic of neutrophils, were detected after exposure to tarin for five days (Figure 5A–C). 

Cytoplasm vesicles were apparently larger and more numerous in cells cultivated with encapsulated tarin when compared to free tarin or control cells (Figure 5A–C) after exposure to tarin for five days. 

#### 2.3.2. In Vitro Antitumoral Activity of Free and Encapsulated Tarin

**Figure 6:**
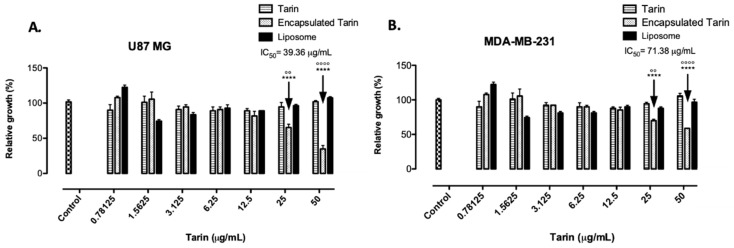
Toxicological effects of free or encapsulated tarin on human tumoral cells. Viability of human (**A**) glioblastoma U-87 MG cell line and (**B**) adenocarcinoma MDA-MB-231 cell line. Non-treated wells, filled with cells and medium, were used as control. To unify the units represented in x axis, tarin concentrations were chosen as reference. Liposome and encapsulated tarin groups are at the same liposome concentration differing only by the presence of tarin. **** *p* < 0.0001 compared to control; °° *p* < 0.01 and °°°° *p* < 0.0001 compared to free tarin.

The antitumoral activity of free and encapsulated tarin in comparison with empty liposomes was tested against human glioblastoma U-87 MG and human breast adenocarcinoma MDA-MB-231 cell lines. Tumoral cells were cultivated in the presence of increasing concentrations of free and encapsulated tarin ranging from 0.78125 to 50 µg/mL for 24 h (Figure 6A,B).

Both lineage cells were dose-dependently inhibited by encapsulated tarin, exhibiting an IC_50_ of 39.36 µg/mL and 71.38 µg/mL for glioblastoma and breast cancer cells, respectively. Glioblastoma cells were 65% and 35% susceptible to encapsulated tarin at 50 and 25 µg/mL, respectively (Figure 6A). Similarly, using the same doses of encapsulated tarin, breast cancer cells were 41% and 35% inhibited when compared to the control group. In contrast, free tarin did not affect tumoral cell growth at either dose after 24 h of exposure (Figure 6B).

## 3. Discussion

Liposome encapsulation of bioactive agents has been proven a successful drug-carrier system due to certain important features, such as biocompatibility with cellular membranes and the ability to incorporate hydrophobic or hydrophilic molecules and enhance therapeutic indices. The use of liposome-encapsulated products has been previously established for different purposes, including drug, biomolecule, and gene delivery [25,26]. Since a drug delivery system can be restricted by physical and chemical instabilities, the preparation of liposomal formulations is a critical step that requires special attention in order to achieve optimal efficiency.

Herein, liposomes preparation was performed according to two distinct protocols, in order to select the most efficient one [20,21]. Both methodologies were adapted to produce nanoliposomal vesicles composed of DOPE (18:1), a neutrally charged phospholipid as the main liposome component, followed by CHEMS, a cholesterol component which fluidizes the membrane, providing additional stability by minimizing bilayer permeability and contributing with cohesive strength [27,28], and PEG, which reduces macrophage recognition, preventing liposomes from a quick clearance from the blood stream, and able to retain tarin for an extended period of time during circulation with no significant loss [29]. Stable liposomes were successfully produced, presenting a typical round shape, ideal size, and polydispersity index as evidenced by DLS analysis (Figure 1). The modified dos Santos Ferreira et al. [21] protocol was used, a higher entrapment efficiency was achieved and this formulation, identified herein as A1, was used as the standard protocol and was further modified, leading to four additional formulations (A1, A2, B1, B2, and C) (Figure S1). The Z average, PdI and capability to hold entrapped tarin for each formulation were monitored for 40 days, in order to select the best liposomal preparation (Figure 2A–C). Formulation A1 was produced without PEG, using two different organic solvents for lipid solubilization, chloroform [21], and methanol [20], as well as different sonication times, initial tarin loads and exposure times for tarin entrapment. The PEG coating was shown to be essential to control nanoparticle size, entrapment efficiency, homogeneity, and stability (Figure 2A–C). Changing solvents, replacing methanol by chloroform, which could be less harmful to the environment, did not lead to remarkable differences in size, homogeneity or entrapment efficiency. However, this change affected stability, leading to higher tarin leakage during the 40-day experiment (Figure 2C). Variations in sonication, exposure time for tarin entrapment, and initial tarin load did not affect physical liposome characteristics. 

Both phospholipid hydrolysis and oxidation led to instability resulting in increased leakage rates of the encapsulated agent or vesicle aggregation, which leads to increased size [30,31]. Vesicle aggregation may have occurred in C formulations, since these presented the highest variations in size and PdI throughout the 40 days of storage at 4 °C. Aggregation may be occurred due to the lack of PEG in formulation C. Thus, this formulation was discarded (Figure 2A,B). Encapsulated tarin leakage was also investigated, in order to choose the formulation able to improve liposome-mediated drug delivery, since leakage can decrease the amount of the drug available for delivery, thus defeating the ability for efficient drug entrapment [32]. The most significant difference observed in leakage rates was detected in liposomes whose lipids were dissolved in methanol in comparison to those dissolved in chloroform (Figure 2C). Taking into account all liposomal nanocapsule features, the A1 formulation exhibited the ideal composition and matched the constraints to ensure therapeutic efficacy, and was, thus, chosen for subsequent assays. 

Nanoparticles displaying diameters of about 100 nm are able to permeate the endothelial layer of blood vessels, reaching the bloodstream, followed by target tissues, and should be capable to deliver tarin to tissues [33,34]. Their spherical shape and smooth surface, confirmed by scanning electron microscopy, decreases hemodynamic forces, allowing them to easily circulate inside the vessels [35]. Different-sized nanocapsules were observed and evidenced by scanning electron microscope images and DLS measurements (Figure 1 and Table 1), corroborating previous results, since liposome production usually displays polydispersity in vesicle diameters, and the ability to finely adjust their geometry and size may be critical for the development of efficient carriers [35].

A controlled tarin release was achieved in a certain pH range, which is significantly advantageous, since liposomes could then be applied for several purposes, as release of encapsulated material could occur, leading to different traits and/or intracellular compartments (Figure 3). The release of tarin at pH 7.4 indicates that it can circulate in the bloodstream or be absorbed by any target tissue, release at pH 6.6 would allow tarin to reach the interstitium of a tumoral mass, and at pH 5.6 or 4.6 the liposome could be phagocyted and released at any stage from the endosome to the lysosome [36]. Although a preference for a specific pH was not clearly demonstrated, after 6 h the half-time releases differed considerably between the different assayed pH. Thus, controlled tarin release might be promoted by a combination effect of temperature and pH, since tarin nanocapsules stored at 4 °C exhibited no significant leakage for 180 days. Based on these observations, further experiments are still necessary to establish the releasing mechanism of tarin nanocapsules.

Liposomal tarin nanocapsule toxicity testing is an essential step for the development of a new potential chemotherapeutic adjuvant to guarantee minimal injury risks for healthy tissues. Free or encapsulated tarin was not toxic to healthy mice bone marrow and L929 fibroblast cells at all assessed doses (0.78125–100 μg/mL) (Figure 4). Thus, at the dosage range tested herein, liposomal tarin nanocapsules could be safely applied both in vitro and in vivo for further experimental evaluations, with the potential to increase their therapeutic efficiency while being protected from cellular proteolytic systems. A proliferative effect for both cell types cultured for 24 h was observed in some free tarin doses, indicating a quicker effect when compared to the encapsulated form. Tarin nanocapsules seems to require an additional time to promote a similar effect, since they were shown to release lectin in constant, but low, doses. This observation was corroborated by the optical microscopy analysis, which revealed the presence of fibroblast-like and/or large rounded cells, characteristics of stromal and progenitor cells, in mice bone marrow cell cultures treated with free or encapsulated tarin after 14 days of culture. Moreover, cytospin slides of the cultured cells evidenced the presence of cells displaying morphological granulocyte and monocyte characteristics after five days of tarin treatment. Merida et al. [17] demonstrated that tarin was able to maintain hematopoietic progenitors and promote granulocyte repopulation (GR1 + cells) in in vitro mice bone marrow cell culture, corroborating the observations described herein and indicating that encapsulated tarin may present a similar potential to stimulate hematopoiesis. However, further studies using cell molecular markers should be performed to determine the immunomodulatory potential of liposomal tarin nanocapsules.

Additionally, several vesicles were observed in the cytoplasm of mice bone marrow cells treated with liposomal tarin nanocapsules, indicating high intracellular activity and constant production of molecules by the endoplasmic reticulum, evidenced by intracellular vesicle formation (Figure 5C). This observation corroborates the hypothesis considered by Merida et al. [17], who states that free tarin may activate transcriptional factors in these cells and stimulate molecule production, such as cytokines or growth factors, which could trigger the multiplication or differentiation of progenitor cells in the bone marrow environment. Although evidence indicates encapsulated tarin immunomodulatory activities, additional studies must be performed in order to establish its mechanism of action and identify the affected bone marrow cell population.

Considering the lack of toxicity to healthy cells associated to an immunostimulatory effect, liposomal tarin nanocapsules exhibited promising results inhibiting the proliferation of human glioblastoma and mammary adenocarcinoma cell lines. These cancer cells lines presented sensitivity to encapsulated tarin comparable to usual chemotherapeutic drug effects. Concerning glioblastoma, the determined IC_50_ for encapsulated tarin (39.36 μg/mL) was equivalent to 65% of cisplatin (29 μg/mL) and 82.2% of temozolide (33.40 μg/mL) inhibition growth effects (Table 2) [37,38]. On the other hand, breast adenocarcinoma cells displayed a less favorable panorama when compared to literature results. Liposomal tarin nanocapsules inhibited breast cancer cell proliferation, displaying an IC_50_ of 71.38 μg/mL, while doxorubicin and cisplatin exhibited IC_50_ of 0.50 and 2.30 μg/mL, respectively [39,40] (Table 2). It should be considered that conventional chemotherapeutic drugs are highly cytotoxic to healthy cells since their effective dose against tumoral cells is usually close to the cytotoxic doses for healthy cells, meaning they display a low selective index, the ratio between the CC_50_ (the concentration required to cause 50% cytotoxicity) and IC_50_ (the concentration required to cause 50% inhibition). In this study, the CC_50_ value for healthy mice cells was unable to be determined, as no cytotoxic effect was observed up to 100 µg of tarin. It is possible that encapsulated tarin presents a higher selective index compared to doxorubicin or cisplatin. These tarin nanocapsule properties would ensure a safer application of this composite at effective doses with minimal adverse effects to healthy tissues. On the other hand, exposure time should also be considered, since antitumoral activity was evaluated after only 24 h. To answer this question, further cytotoxicity experiments should be performed with mice healthy cells using higher tarin doses and longer exposure tarin times in order to determine the selective index.

Interestingly, free tarin failed to exhibit cytotoxicity to the assessed cancer cell lines, since tumoral cells were 100% resistant to the free form of tarin (Figure 6A,B). It is known that the taro lectin recognizes and binds to high mannose *N*-glycans that are a part of the Lewis Y/CD174, H2/CD173, and CA125 antigens, commonly found in human cancer cells [11,16]. Additionally, free tarin has been reported to exhibit a modest response to antitumoral cells, including the murine mammary tumor cell line 66.1 [14,15]. Although a direct comparison cannot be carried out because Kundu et al. [15] did not specify the tarin concentration used in antitumoral assays, it is possible that the free tarin concentrations used herein were not enough to exert the antitumoral effect but that nanoencapsulation of tarin in liposomes may contribute to promote tarin internalization, enhancing intracellular concentrations, triggering stronger antitumoral activity, and potentiating the inhibition mechanism. However, tarin molecular mechanisms on cancer cells has not been evaluated. Further studies should be carried out to determine if tarin could act on cancer cell surfaces through specific carbohydrate-binding, as hypothesized in a previous study, or if the lectin could be internalized, both in its free and encapsulated form [16]. A combination of both mechanisms should also be considered. 

In addition, according to Merida et al. [17], when tarin was administered combined with cyclophosphamide (CY), a currently applied breast cancer drug, a protective effect against CY cytotoxicity was observed, decreasing the frequency of micronucleated erythrocytes and also displaying immunostimulatory potential Thus, even if tarin encapsulation does not contribute to pharmacological effects comparable to doxorubicin and cisplatin in breast cancer cells (MDA-MD-231) proliferation, it could minimize cyclophosphamide cytotoxicity and protect progenitor hematopoietic cells, allowing for faster recovery.

Due to the successful and non-toxic tarin-liposome encapsulation and its activity against human tumoral cells, tarin may be considered a promising pharmacological agent if the methodologies used for tarin obtainment and encapsulation are reproductive and the yield compatible with scale-up the drug preparation. Considering a standard procedure, 100 g of taro corms—smaller than a tennis ball can yield about 300 mg of purified tarin which, after liposomal encapsulation could result in liposomal tarin nanocapsules sufficient to be used in 3000 in vivo murine model assays or 12,000 in vitro culture cell lineage assays. In addition, the fact that taro is cropped throughout the entire year in tropical or subtropical zones, which include large South America, Africa, and Asia areas, located between the Tropics of Cancer and Capricorn, should also be considered. Taro is consumed as a supplementary carbohydrate source in these areas and its extract has been used by indigenous populations for medicinal purposes since ancient times.

Although further pre-clinical trials may be performed, the results reported herein have encouraged us to invest in the development of liposomal tarin nanocapsules to be tested as an adjuvant candidate presenting immunomodulatory and antitumoral activities in a tumor-bearing murine model considering a classical chemotherapy regimen.

## 4. Material and Methods

### 4.1. Tarin Purification

*Colocasia esculenta* corms were purchased at a local market in the Niterói municipality (22°52′51″ S, 43°6′15″ W), Southeastern Brazil. The crude taro extract was obtained according to Roy et al. [41], with modifications, and fractionated using the affinity chromatography resin Cibacron Blue 3G-A (Sigma-Aldrich Co, St. Louis, MO, USA), as described by Pereira et al. [10]. In order to reduce interferences in subsequent in vitro and in vivo experiments, the Tris-HCl present in the purified fraction containing tarin was removed by dialysis (Fisherbrand, Pittsburgh, PA, USA) against water at 8 °C for 18 h under constant stirring and lyophilized (Labconco, Kansas, MO, USA) for storage. Protein quantification in the crude extract was performed by the Peterson [23] method, using bovine serum albumin (BSA) as standard.

### 4.2. Liposomal Nanocapsules Preparation

Liposomes were obtained by modifying the protocols described by dos Santos Ferreira et al. [21] and Andrade et al. [20]. Liposomes were prepared by dissolving the lipid components DOPE (1,2-dioleoyl-sn-glycerol-3-phosphoethanolamine) (Lipoid GMBH, Luidwigshafen, Germany), MPEG 2000-DSPE 1,2-distearoyl-sn-glycero-3-phosphoethanolamine-*N*-[amino(polyethylene glycol)-2000] (ammonium salt) (Lipoid GMBH) and CHEMS (cholesterylhemisuccinate) (Sigma-Aldrich Co) (5.7:3.8:0.5 μmol of lipids) in a chloroform solution under constant stirring at 150 rpm for 15 min. The organic solution was then removed by evaporation under reduced pressure for 25 min at 40 ± 1 °C and constant stirring at 120 rpm. The dry lipid film was rehydrated to reach a 0.01 M concentration in an aqueous phase consisting of 0.3 M ammonium sulfate (pH 7.4) containing tarin at 1 mg/mL, followed by incubation for 12 h at 4 °C. Then, the suspension was maintained under magnetic stirring for 40 min and the large unilamellar vesicles formed were sonicated for 1 min, followed by a 12-cycle extrusion through a polycarbonate 0.2 μm pore membrane. Unencapsulated and encapsulated tarin were recovered by ultracentrifugation carried out at 150,000× *g* at 4 °C for 90 min using an Optima L-90k ultracentrifuge (Beckman Coulter, Brea, CA, USA). The pellet containing encapsulated tarin was suspended in a HEPES buffered saline solution (HBS) at pH 7.4 and the supernatant containing unencapsulated tarin was quantified by Peterson’s method [23] to avoid lipid interference. Entrapment efficiency was determined by the ratio between the amount of unencapsulated tarin and the original amount of tarin used in the encapsulation assay [22].

The liposome preparation protocol was subjected to some variations in order to optimize stability and entrapment efficiency conditions. Different organic solvents, sonication times, initial tarin loads, and times for tarin entrapment were tested (Figure S1).

### 4.3. Morphological Liposome Characterization

Liposome characterization was performed according to Murtey and Ramasamy [42]. Liposomes were attached to microscope slide coverslips, previously coated with poly-l-lysine, and fixed in 4% glutaraldehyde prepared in 0.1 M phosphate buffer pH 7.2, at 4 °C for 48 h. The coverslips were then washed three times for 5 min with the same phosphate buffer. Fixed samples were then dehydrated as follows: 35% ethanol 1× for 15 min, 50% ethanol 1× for 15min, 75% ethanol 1× for 15 min, 95% ethanol 2× for 15 min and absolute ethanol 3× for 20 min. Subsequently, samples were chemically dried by immersion in 1–2 mL of hexamethyldisilazane (HMDS) for 10 min, twice, and left in a desiccator at room temperature overnight. Dried samples were then mounted on a sample stub with a double-sided sticky tape, sputtered with gold and visualized using a JEOL JSM-6460LV scanning electron microscope (SEM) (JEOL, Peabody, MA, USA) at 20kV.

### 4.4. Encapsulated Tarin Stability Determination

Size distribution and the polidispersity index (PdI) of liposomal tarin nanocapsules stored at 4 °C for 180 days were assessed by dynamic light scattering (DLS) (Zetasizer Malvern Panalytical, Almelo, NLD).

Nanocapsule tarin leakage was quantified by Peterson’s method [23] during 40 days of storage at 4 °C.

### 4.5. Kinetic Release of Encapsulated Tarin

The influence of time, temperature and pH on tarin release was determined according to the protocol described by Deniz et al. [43]. A liposome suspension containing tarin was diluted (1:10) in phosphate buffered saline at pH 4.6, 5.6, 6.6, and 7.4., followed by incubation at 36 °C under constant gentle agitation. Aliquots (100 μL) were collected at 0, 1, 2, 3, 4, 5 and 6 h to determine the amount of released tarin, applying Peterson’s method [23].

### 4.6. Pre-Clinical In Vitro Tests

#### 4.6.1. Animals

Adult isogenic male C57Bl/6 mice (aged 10–16 weeks) were provided and maintained at the Animal Center Laboratory (NAL), belonging to the Universidade Federal Fluminense (UFF), Rio de Janeiro, Brazil.

The research protocol was approved by the Universidade Federal Fluminense Ethics Committee in Animal Use (CEUA), under No. 821/2016.

#### 4.6.2. Tarin Cytotoxicity on Healthy Bone Marrow and Fibroblasts Cells

Tarin cytotoxicity was assessed by the median inhibitory dose, capable of killing 50% of cultured cells. Healthy mouse bone marrow and L929 cell lines (Sigma-Aldrich Co) were seeded in 96-well polystyrene microplates at a concentration of 5 × 10^5^ cells/well and 1.5 × 10^5^ cells/well, respectively, in 100 μL of RPMI-1640 media (Sigma-Aldrich Co), supplemented with 10% fetal calf serum (FCS), 2 mM l-glutamin, 5 × 10^5^ M2-mercaptoethanol, and incubated at 37 °C in a humidified atmosphere containing 5% CO_2_. After 24 h incubation, the time required for cell adherence to the plate, different free or encapsulated tarin concentrations (from 0.78125 to 100 μg/mL) and empty liposomes were added to a final volume of 100 μL. The positive controls comprised cells cultivated in culture media, while 12.5% sodium azide (25 μL) was added to the negative control wells. Cells were incubated for an additional 24 h and cell viability was assessed by the colorimetric assay using resazurin as the indicator. A 20 μL aliquot of resarzurin 125 μg/mL was added to each well, followed by 4 h of incubation. Fluorescence intensity was determined using a SpectraMax Microplate Reader M4 (Molecular Devices, Sunnyvale, CA, USA) at 530 nm (excitation filter), 570 nm (cutoff), and 586 nm (emission filter) wavelengths.

#### 4.6.3. Morphology of Bone Marrow Cells Treated with Free and Encapsulated Tarin

Cells were cultured (2 × 10^6^ cells/mL) in RPMI-1640 media (Sigma-Aldrich Co), supplemented with 10% fetal calf serum (FCS), 2 mM l-glutamin, 5 × 10^−5^ M 2-mercaptoethanol and 20 µg/mL gentamicin, in the presence or absence of 20 µg/mL tarin, at 37 °C in a humidified atmosphere containing 5% CO_2_, for 14 days. Media were replaced every five days. Cell samples were collected on days 0, 5, and 14, and transferred to glass slides by centrifugation (284× *g* for 10 min at room temperature) using a Cytopro 7620 centrifuge (WESCOR Inc, Logan, UT, USA). Cells were analyzed after staining by the May–Grunwald–Giemsa method under an optical microscope (Olympus BX41, Shinjuku, Tokyo, Japan) [44]. Photomicrographs of the cultures were acquired under an inverted-phase microscope Zeiss Telaval 31 (Carl Zeiss Co., Oberkochen, Germany) and occupied area was determined using the ImageJ software (version, version 2, Wayne Rasband, Bethesda, MD, USA) [45].

#### 4.6.4. Evaluation of Antitumoral Tarin Activity

The viabilities of the human cancer cell lines MDA-MB-231 (ATCC^®^ HTB-26™) (breast adenocarcinoma) and U-87 MG (ATCC ^®^ HTB-14™) (glioblastoma) were determined by the redox resazurin method described in Section 4.6.2. Cells (1.5 × 10^5^ cells/well) were seeded in 25 cm^2^ culture bottles, containing Dulbecco’s modified Eagle medium (Nutrient Mixture F-12 - DMEM/F-12) (Sigma-Aldrich Co), supplemented with 10% fetal calf serum (FCS), 2 mM l-glutamin, 5 × 10^5^ M2-mercaptoethanol. Culture bottles were incubated at 37 °C in a 95% humidified atmosphere containing 5% CO_2_ until a semiconfluent monolayer was achieved.

Cells from the semiconfluent monolayer were detached from the culture bottles with soft movements after the addition of 2 mL of a solution containing 1 mL 20% trypsin, 0.5 mL 10% ethylenediaminetetraacetic acid (EDTA), and 3.5 mL PBS. Cell suspensions were then centrifuged at 200× *g* for 7 min and pelleted cells were suspended in DMEM medium followed by cell counting using an optical microscope in a Neubauer hemocytometer (Laboroptik, Lancing, England, UK). Cancer cells at 5 × 10^5^ cells per well, at a final volume of 100 μL, were cultivated in 96-well culture microplates containing DMEM medium and the cytotoxicity evaluation procedure was performed as described previously.

The units displayed in the *x*-axis were standardized per tarin concentration (Figure 6). For this purpose, empty liposomes and encapsulated tarin were prepared using the same parameters, composition, and amount of components. The final preparation was suspended in equal volumes (3 mL) and the serial dilution of encapsulated and empty liposomes was prepared using a similar initial volume (100 μL) to ensure that both samples presented the same liposome concentration, differing only in the presence of tarin. Based on this, liposome concentration was similar in both preparations with and without tarin.

### 4.7. Statistical Analyses

The results were compared through an analysis of variance (ANOVA) followed by multiple comparisons by Tukey’s method. The GraphPad Prism software (version 7, GraphPad, San Diego, CA, USA) was used.

## 5. Conclusions

The methodology described herein allowed for the reproducible production of spherical, smooth-surfaced, and nanomeric (<0.2 nm) liposomal capsules that exhibit high tarin entrapment efficiencies and low leakage rates under storage conditions. Liposomal tarin nanocapsules exhibited no toxicity to healthy mice bone marrow and L929 cells and inhibited the proliferation of MDA-MB-231 and, to a higher extent, U-87 MG cell lines, indicating an improvement of the pharmacological ability of encapsulated tarin compared to its free form. Healthy mice bone marrow cells treated with free and encapsulated tarin also indicates a possible immunomodulatory effect, which could be useful to boost the host immune system. Liposomal tarin nanocapsules seem to be a promising anti-cancer candidate, obtained from a food matrix and exhibiting two of the most valuable characteristics for any anticancer drug, namely anti-tumor efficacy and low toxicity to healthy cells, enabling it as a putative composite to be associated as an adjuvant to traditional chemotherapy.

## Figures and Tables

**Table 1:** Stability of liposomal tarin nanocapsules.

Membrane Pore Size	Storage Time Intervals at 4 °C (days)	Size Distribution (nm)	Average Size (nm)	Polydispersity Index (PdI)	Peak (nm)	Entrapment Efficiency
0.2 µm	1	50.75–396.1	154.6	0.168	93.55 ± 38.75	0.83
	10	43.82–396.1	155.0	0.191	78.74 ± 35.19	
	90	50.75–396.1	149.8	0.163	88.21 ± 35.69	
	120	43.82–396.1	149.2	0.192	84.65 ± 34.57	
	150	50.75–396.1	151.3	0.191	39.64 ± 22.36	
	180	58.77–396.1	149.9	0.135	99.12 ± 36.50	

**Table 2:** Single-dose cytotoxicity of conventional drugs and encapsulated tarin to human tumoral cells after 24 h exposure.

Tumoral Cell Lines	Antitumoral Molecules	IC_50_ (µg/mL)	Reference
U-87 MG	Cisplatin	29.00	[35]
	Temozolomide	33.40	[36]
	Tarin liposomal nanocapsules	39.36	Present study
MDA-MB-231	Cisplatin	2.30	[37]
	Doxorubicin	0.50	[38]
	Tarin liposomal nanocapsules	71.38	Present study

## Supplementary Materials

The following are available online, Figure S1: Preparation of liposomal tarin nanocapsules using different formulations and procedure.
