# Supplementary material for: Liposomal Taro Lectin Nanocapsules Control Human Glioblastoma and Mammary Adenocarcinoma Cell Proliferation
Source: Molecules. 2019 Jan 29;24(3):471. doi: 10.3390/molecules24030471 (PMC6385085; doi:10.3390/molecules24030471)
Supplement: Supplementary file 1 [file molecules-24-00471-s001.pdf]

Supplementary Material

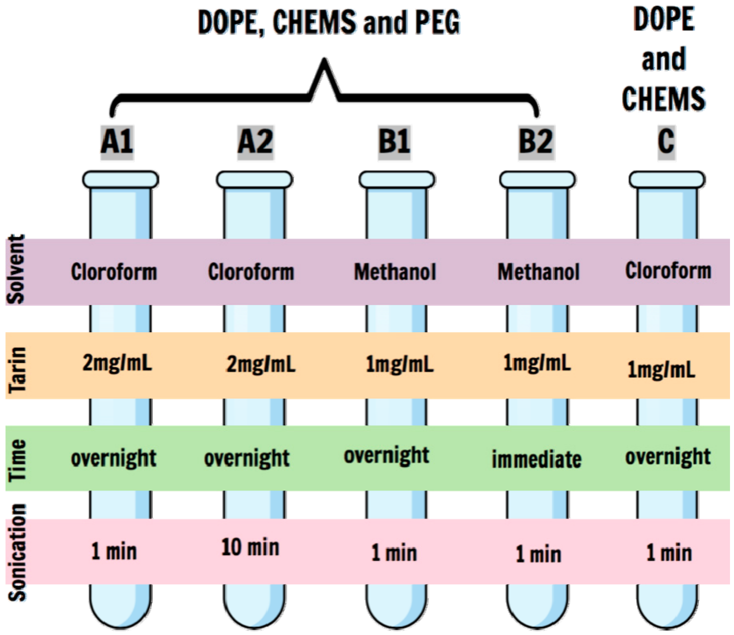

Figure S1. Liposomal tarin nanocapsules were prepared following the dos Santos Ferreira et al. [21] protocol with modifications. The basic formulation (A1) was modified according to type of solvent, initial tarin load, sonication and tarin entrapment time, with or without DSPE-PEG (2000). **DSPE-PEG (2000)** - amine 1,2-distearoyl-sn-glycero-3-phosphoethanolamine-N-[amino(polyethylene glycol)-2000] (ammonium salt); **DOPE -** 1,2-dioleoyl-sn-glycerol-3-phosphoethanolamine; **CHEMS -** cholesterylhemisuccinate.
